# Water Distribution Systems in Pig Farm Buildings: Critical Elements of Design and Management

**DOI:** 10.3390/ani11113268

**Published:** 2021-11-15

**Authors:** Stephen Little, Andrew Woodward, Glenn Browning, Helen Billman-Jacobe

**Affiliations:** 1 Asia Pacific Centre for Animal Health, Melbourne Veterinary School, Faculty of Veterinary and Agricultural Sciences, and National Centre for Antimicrobial Stewardship, University of Melbourne, Parkville, VIC 3010, Australia; glenfb@unimelb.edu.au (G.B.); hbj@unimelb.edu.au (H.B.-J.); 2 Melbourne Veterinary School, Faculty of Veterinary and Agricultural Sciences, University of Melbourne, Parkville, VIC 3010, Australia; Andrew.Woodward@canberra.edu.au

**Keywords:** drinking water, water distribution system, hydraulic performance, flow rates, pig drinkers, water sanitization, biofilms, water medication, antibiotic, antimicrobial resistance

## Abstract

**Simple Summary:**

The piped water systems within buildings on pig farms provide pigs with continuous access to drinking water, and on many farms are also used for short periods to medicate growing pigs with antibiotics to help keep them healthy and productive. We surveyed managers of 25 medium to large pig farms across eastern and southern Australia to investigate critical elements of the design and management of water systems that impact water provision to pigs. We found wide variation in the configuration, length, and pipe materials and diameters of water systems in buildings across farms. In many buildings, main pipelines were larger in diameter than required. While this helps ensure that drinkers always provide plenty of water to pigs, it means water flows through pipes very slowly. We also found that in many buildings the number of pigs per drinker was above the recommended maximum, cleaning and disinfection of water systems was not done on many farms, and few managers were aware of the risks to water quality and pig health. We have identified important aspects of water provision to pigs for which recommendations could be added to industry guidelines used by pig farm managers.

**Abstract:**

Drinking water distribution systems (WDSs) within buildings on pig farms have critical elements of their design and management that impact water provision to pigs, water quality, the efficacy of in-water antimicrobial dosing, and, thus, pig health and performance. We used a mixed-methods approach to survey managers of 25 medium to large single-site and multi-site pig farming enterprises across eastern and southern Australia. We found wide variation in the configuration (looped or branched) and total length of WDSs within buildings across farms and in pipe materials and diameters. Within many conventional buildings and some eco-shelters, WDSs were ‘over-sized’, comprising large-diameter main pipelines with high holding volumes, resulting in slow velocity water flows through sections of a WDS’s main pipeline. In over half of the weaner buildings and one-third of grower/finisher buildings, the number of pigs per drinker exceeded the recommended maximum. Few farms measured flow rates from drinkers quantitatively. WDS sanitization was not practiced on many farms, and few managers were aware of the risks to water quality and pig health. We identified important aspects of water provision to pigs for which valuable recommendations could be added to industry guidelines available to pig farm managers.

## 1. Introduction

**Figure 1:**
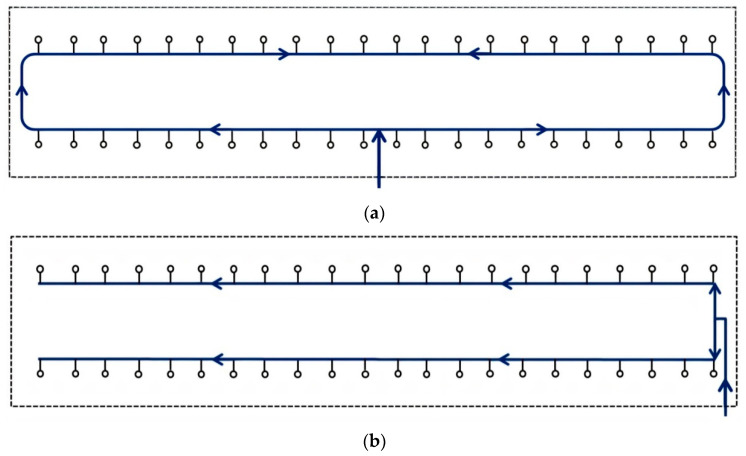
Examples of two configurations of a drinking water distribution system within a conventional pig building (concrete/slatted floors): (**a**) looped, (**b**) branched. Main pipelines are represented by blue solid lines. Arrows indicate the direction of water flow. Drinkers are represented by open circles. Walls of the building are represented by dashed lines.

A pig farm’s water distribution system (WDS) transports drinking water through pipes from one or more sources to each building accommodating pigs and distributes it throughout each building to drinking appliances (drinkers) in each pen. The WDS is pressurized by one or more pumps or by gravity from an elevated water source or storage facility. Within each pig building, the main pipeline has either a looped or branched configuration (Figure 1).

WDSs in pig buildings are demand-based systems, with pigs able to access water from drinkers ad libitum. Each pig must consume between 60 and 117 mL/kg bodyweight (BW) each day to maintain a balance between its bodily water inputs and outputs [1,2,3]. To enable growing and breeding pigs to drink to satiety without restriction, recommended water flow rates from drinkers are 0.25–0.5 L/min for weaner pigs (5–25 kg BW) and 0.5–1 L/min for grower/finisher pigs (20–130 kg BW) [4]. A building’s WDS must be able to sustain these flow rates 24 h per day from every drinker when the building is at maximum capacity and pigs are approaching their final bodyweight, regardless of the drinker’s distance from the point where the pipeline enters the building. If a building’s WDS has been poorly designed or modified, or if one or more sections of pipe have become occluded by biofilms and sediments, this may cause spatial and/or temporal variation in the access of pigs to drinking water. If the variation is sufficient to impose a substantial degree of restriction on pigs’ water access, this may lead to competition for water between animals in each pen, thereby contributing to between-animal variability in drinking and feeding patterns, in daily consumption of feed and water, and in the rate of weight gain. If restriction of pigs’ water access is severe, it may lead to increased aggressive interactions between pigs, compromising pig welfare [5,6,7].

Water flow through the building’s WDS is very dynamic. Flow rates within each pipe section and the residence time (‘age’) of water at each drinker may change markedly from hour to hour each day, depending on characteristics of the WDS and pigs’ water demand, which is a function of the number of pigs in the building, their bodyweight, and their drinking patterns [8]. Important design characteristics of a WDS are its configuration (looped or branched); the length, diameter, and smoothness of the internal surface of each section of pipe; the system’s head pressure; and the number of bends and constrictions or expansions in the diameter of pipes along the system, which result from the use of fittings [9].

On many pig farms in Australia and other countries, WDSs in buildings accommodating weaner and grower/finisher pigs serve a second purpose, as systems for mass medication of pigs with antimicrobials for metaphylaxis and to treat clinical disease caused by bacterial pathogens [10,11,12,13]. Other additives, including vaccines, parasiticides, organic acids, electrolytes, minerals, vitamins, amino acids, sweeteners, direct-fed microbials, essential oils, and potential new therapeutic products, such as bacteriophages, may also be administered to pigs through WDSs [11]. A water-soluble antimicrobial product may be administered into a building’s main water line through a proportional dosing pump or header tank, and the hydraulic performance of the building’s WDS determines the time course of antimicrobial drug concentration in water available to pigs at drinkers in each pen during and after the dosing event. Differences in the antimicrobial concentration in water delivered to pigs at drinkers in each pen over time by the WDS are a source of between-animal variability in the in-water dosing process and may affect the number of pigs that have systemic levels of the antimicrobial that are sufficient to successfully eliminate or substantially reduce numbers of the target pathogen and achieve high clinical efficacy, while minimizing selection for and propagation of resistant pathogens [8,14,15].

We surveyed the managers of medium to large single-site and multi-site pig farming enterprises across eastern and southern Australia with the aim of investigating critical elements of the design and management of WDSs in weaner and grower/finisher buildings that influence the quantity and quality of water provided spatially and temporally to growing pigs and the efficacy of in-water dosing when practiced.

Mixed-methods research approaches originated in the field of social and behavioral sciences [16]. Over the past 20 years, as the advantages of collecting, analyzing, and interpreting quantitative and qualitative data in a single study to investigate a research question have been realized, mixed-methods research has become well accepted and commonly used in many other fields, including business management, health and medical sciences, and veterinary and agricultural sciences [17]. Our survey used a mixed-methods approach with a sequential explanatory design [18] with the quantitative and qualitative data having equal weighting [19]. The mixed-methods approach enabled us to confirm findings using different methods, adding depth and confidence in the conclusions. The authors’ experience was that the quantitative data were highly informative for the subsequent interview, enabling questions to be tailored to obtain richer, more detailed responses.

## 2. Materials and Methods

A purposive method was used to obtain a sample of farm managers of medium to large single-site and multi-site pig farming enterprises across Australia. To be eligible to participate in the survey, a person was required to be a pig farm manager responsible for management of the water system and in-water antimicrobial dosing of growing pigs. Their farm had to have operated for at least 6 months, have more than 500 weaner and grower pigs, have participated in the Australian Pork Industry Quality Assurance Program [20], have reared growing pigs indoors (in buildings with solid/slatted/mesh floored pens or ‘eco-shelters’ with straw-floored pens), and have water-medicated weaner and/or grower/finisher pigs with antimicrobials for metaphylaxis or treatment of bacterial diseases. Demographics of the 25 pig farm managers who participated in the survey and characteristics of the farms and weaner and grower/finisher buildings on them are presented in Appendix A.

Data were collected on aspects of farm WDSs as part of a larger survey that also examined in-water dosing systems and medication programs. Quantitative data were collected using an online questionnaire completed by each farm manager (available in Supplementary Materials). Qualitative data were then collected in an individual, semi-structured interview with each manager. The questionnaire was designed to provide an understanding of variability across farms about the features of the buildings in which growing pigs were reared and about key aspects of the farms’ WDSs including farm water sources and storage facilities, water lines to buildings and within buildings, drinkers for pigs, monitoring of water supply to pigs and water quality, and biofilm management. Participants were asked to complete the questionnaire within 2 weeks of receipt using a web link and they were interviewed by the lead author (S.L.) within 4 weeks of completing the questionnaire, using an interview guide (available in Supplementary Materials). The study was conducted with the approval of the University of Melbourne Human Ethics Advisory Group (I.D. No. 1853192.1) and was conducted in compliance with its conditions.

Questionnaire responses captured in REDCap from each participant were exported into Excel, de-identified, and then analyzed using the R statistical program [21]. Data were visualized using ggplot. Interviews were transcribed verbatim from audio recordings, de-identified, entered into the qualitative data analysis software package NVivo version 12 (QSR International Pty. Ltd., Melbourne, Victoria, Australia), and openly coded and analyzed using qualitative data analysis principles and thematic analysis [22]. Transcripts were coded manually. The final coding framework used in NVivo is available in the Supplementary Materials. Selected comments made by participants in the interviews are included in the Results section to illustrate the diverse points of view of the participants.

## 3. Results

Twenty-eight pig farm managers were contacted and 25 agreed to participate in the study. The participants’ farms were located in South Australia, Victoria, New South Wales, and Queensland and the majority of the farm managers had been working in the industry for over 10 years. At the time of the study, their farms accommodated 459,167 weaner and grower/finisher pigs. This represents approximately 21% of all growing pigs in Australia at any one time [23]. One farm only housed weaner pigs, while four farms only housed grower/finisher pigs.

### 3.1. Farm Water Sources, On-Farm Water Storage Capacity, and Pipelines to Buildings

Several different sources of stock drinking water had been used across the farms over the past year: bore (8/25), farm dam (6/25), municipal town supply (5/25), river, lake or irrigation channel (3/25), or bore in combination with another source (3/25). Ten farms had used at least one other source of water over the past year. Two farms were totally reliant on rainfall and had run out of water at least once in the past 5 years. Other farms had accessed additional sources of water in recent years to reduce the risk of water shortages. Some farms had established more bores or additional dams.

*We haven’t expanded pig numbers, but our water system, we keep adding to it. We have different sources of water. We have bore water. We have dam water. We have town water and we have desalination water.*

Most managers without access to a pressurized municipal water supply had installed large storage tanks on the farm, which enabled pigs to be supplied with water by gravity for a certain period should an interruption to power supply occur. Most farms had the equivalent of at least 24 h of pig water usage in storage. Four farms had 2–3 days of usage in storage and two farms had 5–7 days of usage in storage. One farm had a diesel back-up generator to ensure water supply during extended power interruptions.

The main pipeline from the water source to the buildings was straight, with branches to each building, on most farms (22/25), and looped around all buildings on three farms. On 13 farms, the main water supply lines to buildings were polyethylene (PE), on eight farms they were polyvinyl chloride (PVC), and on four farms both PE and PVC pipes were used. The internal diameter of the main pipelines to the buildings on farms ranged from 40 mm to 225 mm. On 10 farms they were 50 mm and on nine farms they were 100 mm.

### 3.2. Buildings and WDSs within Buildings

**Figure 2:**
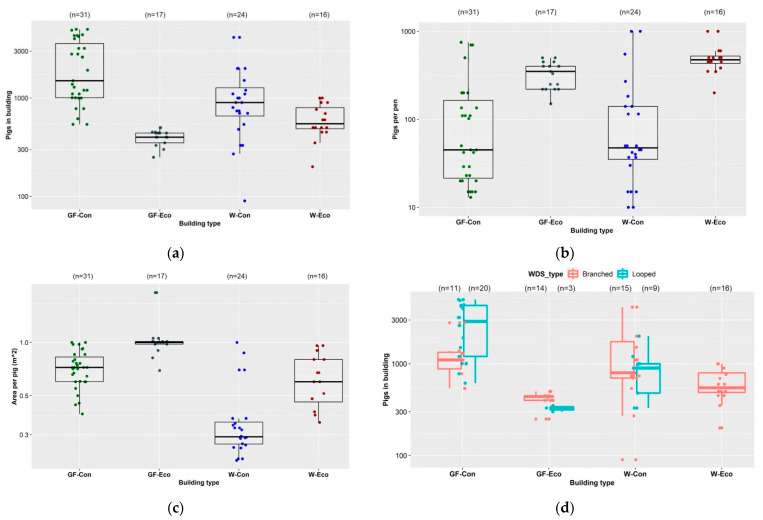
By building type: (**a**) number of pigs in each building, (GF-Con: Q1: 1008, Median: 1500, Mean: 2302, Q3: 3602; GF-Eco: Q1: 350, Median: 400, Mean: 396, Q3: 440; W-Con: Q1:660, Median: 900, Mean: 1196, Q3: 1278; W-Eco: Q1: 488, Median: 550, Mean: 620, Q3: 800); (**b**) number of pigs per pen, (GF-Con: Q1: 22, Median: 45, Mean: 148, Q3: 168; GF-Eco: Q1: 220, Median: 350, Mean: 336, Q3: 400; W-Con: Q1: 35, Median: 48, Mean: 167, Q3: 140; W-Eco: Q1: 433, Median: 475, Mean: 518, Q3: 525); (**c**) surface area per pig (m^2^), (GF-Con: Q1: 0.6, Median: 0.72, Mean: 0.72, Q3: 0.83; GF-Eco: Q1: 0.98, Median: 1.0, Mean: 1.08, Q3: 1.01; W-Con: Q1: 0.6, Median: 0.72, Mean: 0.72, Q3: 0.83; W-Eco: Q1: 0.45, Median: 0.6, Mean: 0.63, Q3: 0.8); (**d**) number of pigs in building by WDS type (branched or looped), (Branched: GF-Con: Q1: 890, Median: 1100, Mean: 1303, Q3: 1334, GF-Eco: Q1: 400, Median: 440, Mean: 411, Q3: 448, W-Con: Q1: 700, Median: 800, Mean: 1371, Q3: 1756, W-Eco: Q1: 488, Median: 550, Mean: 620, Q3: 800; Looped: GF-Con: Q1: 1200, Median: 2905, Mean: 2851, Q3: 4320, GF-Eco: Q1: 315, Median: 330, Mean: 327, Q3: 340, W-Con: Q1: 480, Median: 900, Mean: 904, Q3: 1000). GF-Con = conventional grower/finisher buildings (solid/slatted/mesh floored pens), GF-Eco = grower/finisher eco-shelters (straw-floored pens), W-Con = conventional weaner buildings (solid/slatted/mesh floored pens), W-Eco = weaner eco-shelters (straw-floored pens). All figures are on log10 scale.

The median number of pigs accommodated in conventional buildings with solid/slatted/mesh floored pens was higher than the median number accommodated in eco-shelters with straw-floored pens (Figure 2a). The median number of pigs per pen in eco-shelters was higher than the median number per pen in conventional buildings (Figure 2b). In conventional buildings, the number of pigs accommodated were highly variable, as were the number of pigs per pen. Several farms had combined numerous small pens into fewer, larger pens. The space allowance per pig was greater in weaner eco-shelters than in conventional weaner buildings, and in grower/finisher eco-shelters than in conventional grower/finisher buildings (Figure 2c). The main pipeline of the WDS was looped within 20/31 of the conventional grower/finisher buildings and 15/24 of the conventional weaner buildings included in the survey. Most conventional buildings accommodating more than 1000 pigs had looped WDSs. The WDS was branched within 14/17 of the grower/finisher eco-shelters and all weaner eco-shelters (Figure 2d).

**Figure 3:**
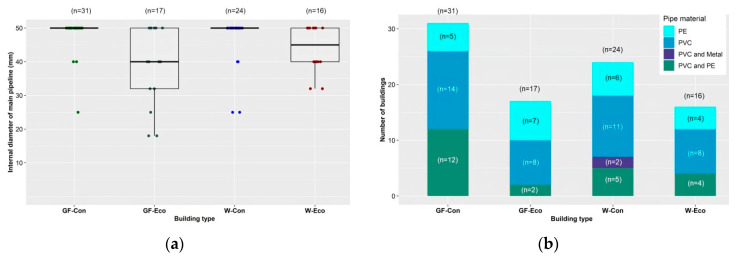
By building type: (**a**) internal diameter of main pipeline (mm), (GF-Con: Q1: 25, Median: 50, Mean: 48.6, Q3: 50; GF-Eco: Q1: 32, Median: 40, Mean: 39.1, Q3: 50; W-Con: Q1: 50, Median: 50, Mean: 47, Q3: 50; W-Eco: Q1: 40, Median: 45, Mean: 44, Q3: 50); (**b**) composition of main pipeline. GF-Con = conventional grower/finisher buildings (solid/slatted/mesh floored pens), GF-Eco = grower/finisher eco-shelters (straw-floored pens), W-Con = conventional weaner buildings (solid/slatted/mesh floored pens), W-Eco = weaner eco-shelters (straw-floored pens). PE = polyethylene, PVC = polyvinylchloride.

Most main pipelines within the conventional weaner and grower/finisher buildings included in the survey were 50 mm in internal diameter. The main pipelines in eco-shelters were more variable in internal diameter (Figure 3a). In each of the four types of buildings, about half the buildings had main pipelines made of PVC. Most other buildings had main pipelines made of PE or a combination of PVC and PE (Figure 3b).

Half of the buildings on the farms were at least 20 years old. Remodeling of existing buildings and construction of new buildings over many years had resulted in modifications and extensions to water pipelines to buildings and within buildings, and these were often considered sub-optimal by the managers of the farms.

*The plumbing system has been added on to or changed every time they made a production change. Everything has just been added on over 35 years, and the whole thing is not ideal.*

Most farm managers (22/25) used the main WDSs within buildings to administer antimicrobials to pigs for short periods for metaphylaxis and treatment. The remaining 3/25 managers had a secondary WDS alongside their main WDS that they used specifically for all in-water dosing. Several farm managers with branched WDSs in buildings expressed uncertainty about their ability to provide pens with equal exposure to an antimicrobial during in-water dosing events. One manager had changed from a branched to a looped system, which he reported reduced the time for water to travel from the dosing pump to each pen from 3 h to 1 h. Two farms were in the process of making simple modifications to building WDSs to transform them into looped systems. Many farm managers reported that several fittings were installed in their buildings’ WDSs: non-return valves to prevent back-flow (13/25), pressure gauges (9/25), pressure regulators (10/25), and water-usage meters (11/25). The final sections of pipe connected to drinkers were often metal, with stainless steel preferred because of its resistance to corrosion. Most conventional grower/finisher buildings had separate water lines with spray nozzles fitted over pens for mitigation of heat stress during periods of hot weather. Most eco-shelters had misting systems fitted to keep pigs cool.

### 3.3. Drinkers for Pigs

Different types of drinkers were provided for weaner and grower/finisher pigs across the farms. Drinkers incorporating a bowl or trough were used in three-quarters (74%) of weaner buildings. Wet/dry feeders were used in weaner buildings on only six farms. Bite and nipple drinkers fixed to pen walls were more commonly used in grower/finisher buildings, with or without wet/dry feeders. Wet/dry feeders were used in grower/finisher buildings on 16 farms. Of those farms, nearly half also provided pigs with water access via bite or nipple drinkers. Three farms used liquid feeding systems. Managers were very aware of the advantage of bowl and trough drinkers (lower wastage) and their disadvantages (poorer hygiene, with the need for more cleaning).

**Figure 4:**
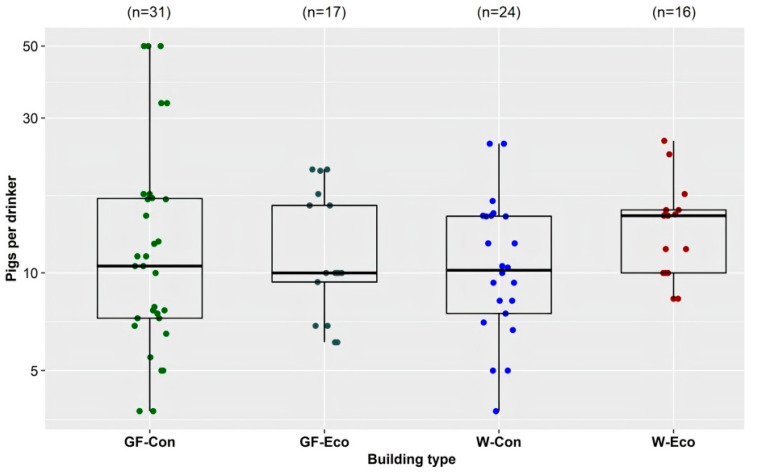
Pigs per drinker by building type (GF-Con: Q1: 7.25, Median: 10.5, Mean: 15.4, Q3: 16.9; GF-Eco: Q1: 9.4, Median: 10, Mean: 12.2, Q3: 16.1; W-Con: Q1: 7.5, Median: 10.2, Mean: 11.4, Q3: 15; W-Eco: Q1: 10, Median: 15, Mean: 14.3, Q3: 15.6). GF-Con = conventional grower/finisher buildings (solid/slatted/mesh floored pens), GF-Eco = grower/finisher eco-shelters (straw-floored pens), W-Con = conventional weaner buildings (solid/slatted/mesh floored pens), W-Eco = weaner eco-shelters (straw-floored pens).

Across the 25 farms, the number of pigs per drinker in conventional buildings and eco-shelters for weaner and grower/finisher pigs varied considerably (Figure 4). The recommended maximum of 10 pigs per drinker for weaner buildings was exceeded in 50% of the conventional weaner buildings and 68% of the weaner eco-shelters surveyed. The recommended maximum of 12–15 pigs per drinker for grower/finisher buildings was exceeded in 32% of the conventional grower/finisher buildings and 35% of the grower/finisher eco-shelters surveyed [24,25,26,27]. Three conventional grower/finisher buildings had 50 pigs per drinker; however, two of these buildings had wet feeding systems in use. Across all farms, regardless of the number of pigs in each pen, at least two drinkers were provided per pen to reduce the number of aggressive interactions between pigs and manage the consequences of a malfunction in the sole drinker in a pen. Many managers had recently installed, or intended to install, extra drinkers in pens to provide pigs with better access to water and provide alternative access should a drinker become blocked. On one farm, small bowl drinkers were fitted temporarily for the first 2 weeks after weaners were placed, to enhance water access while they learned to use the bite nipple drinkers. On another farm, an additional swing drinker was fitted in the center of each pen and was turned on during periods of hot weather to increase water access.

Flow rates from some or many drinkers in each building were checked daily on 19 farms, weekly on two farms, and less frequently on four farms. Only two farms measured flow rates quantitatively using a measuring cup. On all other farms, drinkers were checked using a finger to assess strength of flow or just checked visually. Farm managers were generally content to follow industry guidelines on heights above the ground for nipple drinkers supplying weaner, grower, and finisher pigs [28]. Two managers felt that the drinkers in their weaner pens were too high. One had, therefore, provided additional bowls to provide easier access for young weaners. Height-adjustable drinkers were not used on any manager’s farm.

*I haven’t experimented with it, mainly because there is a code of practice in regards to the height off the ground for varying ages of pigs.*

*I feel the drinker height is too high. Slightly too high for the piglets when they first go in there. It feels like they need to be all dropped down, but that’s a big job in itself.*

Many managers had strong views about where drinkers should be positioned within a pen in order to minimize bullying and maximize the access of pigs to water on hot days. Two managers related drinker position and spacing from feeders to pig performance. However, their views were opposing. One hypothesized that pigs ate more and grew faster if drinkers were close to feeders, while the other had measured improved growth rates when drinkers and feeders were separated, and, as a consequence, had decided to turn off the water supply in the wet/dry feeders and use them as dry feeders. Positioning of bowl drinkers was also related to eliminative behavior by pigs. One manager had recently re-positioned bowl drinkers in their concrete/mesh-floored weaner pens because pigs were fouling them. Another manager intended to re-position bowl drinkers in their straw-floored grower/finisher eco-shelters to reduce fouling.

*If (the drinker) is near feed, they tend to eat and drink and put on more weight with more feed being consumed.*

*My preference is to have the water off to the feeders and have two distinct stations—one drinking station and one feeding station. We did that (and) actually got a one kilo weight gain difference between the traditional and modified systems.*

Only one manager expressed a view about the best orientation of nipple drinkers mounted to pen walls (facing out into the pen vs. parallel to the wall). However, another manager noted that pigs could injure themselves on outward-facing nipple drinkers.

### 3.4. Changes in Daily Water Flow in WDSs as Each Batch of Pigs Is Reared

**Figure 5:**
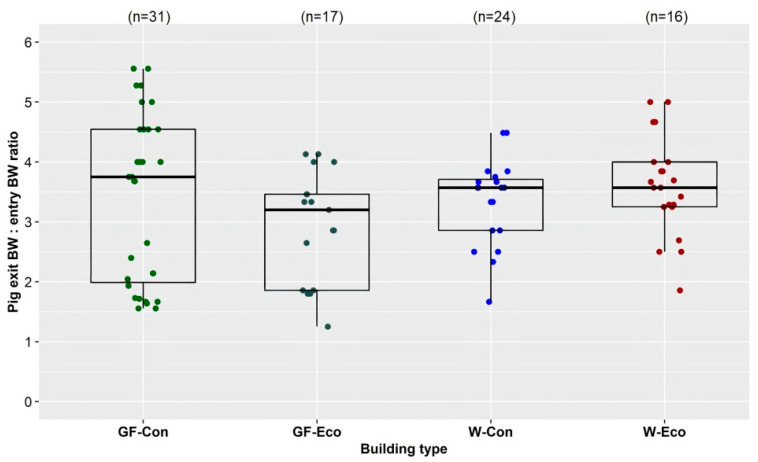
Pig exit bodyweight:entry bodyweight ratio by building type (GF-Con: Q1: 1.99, Median: 3.75, Mean: 3.41, Q3: 4.55; GF-Eco: Q1: 1.86, Median: 3.2, Mean: 2.94, Q3: 3.46; W-Con: Q1:3.21, Median: 3.57, Mean: 3.48, Q3: 3.77; W-Eco: Q1: 3.11, Median: 3.43, Mean: 3.47, Q3: 3.86). GF-Con = conventional grower/finisher buildings (solid/slatted/mesh floored pens), GF-Eco = grower/finisher eco-shelters (straw-floored pens), W-Con = conventional weaner buildings (solid/slatted/mesh floored pens), W-Eco = weaner eco-shelters (straw-floored pens).

Across the 25 managers’ farms, the median weights at which pigs entered and exited the weaner buildings were 7 kg and 25 kg, respectively. The median weights at which pigs entered and exited the grower/finisher buildings were 25 kg and 95 kg, respectively. In buildings of each of the four types, the exit weight of pigs, expressed as a multiple of their entry weight, varied considerably, especially in grower/finisher buildings, while in weaner buildings it was less variable (Figure 5). Many farm managers had set up their pig flow so that pigs were only moved once between weaning and market, from a weaner building to a grower/finisher building. As daily water usage of pigs is proportional to their bodyweight, the total volume of water flowing through a building’s WDS per day would, therefore, be expected to change by about this ratio over the period that each batch of pigs occupied the building.

### 3.5. Monitoring WDS Function and Water Quality

Most farm managers had systems installed to automatically switch spray and mist cooling systems on and off at pre-set temperatures within buildings. However, few managers had systems to monitor and control parameters important to the function of the WDS and dosing system. For example, only nine managers had pressure gauges fitted to pipes. While five managers had alarm systems installed to alert staff of a pump failure or burst water pipe, the other 20 managers relied on staff members to detect such problems. Three managers had increased their pumping capacity to reduce the risk of pump failures and their consequences.

*I live 15, 20 min away. In the middle of summer on a weekend you’ve got to come out and have a look at the pumps. If you could dial up on your phone and check that the pressure gauges are fine or that the tank is full—there’s no need to go there.*

*We always had an issue of blowing water pipes, creating havoc for us. So we changed over to variable speed drive pumps.*

*We run two pumps in our dam. They continually alternate, so we’re getting the same amount of hours on each pump. If I have one pump break down, I’ll just run the other one on manual.*

Fifteen managers conducted water quality analysis at least once per year, while the other 10 managers either never conducted water quality analysis or only did so sporadically. Most managers referred to the quality of water at the source rather than at the point of consumption by pigs. Managers were mainly concerned about chemical quality parameters (e.g., total dissolved salts) and possible blockage of drinker nipples because of accumulation of sediments, algae, or, on some farms, small animals, such as small fish, crustaceans, worms, and snails. One manager used a reverse osmosis system to improve the quality of drinking water for the pigs. Three managers using bore water blended it with another water source to ensure that salt levels were not excessive. Three managers expressed concerns that elevation in the temperature of water in pipes during hot summer days may affect the water consumption and performance of the pigs. On one of these manager’s farms this was due to the use of black PE pipes running along the surface of the ground alongside buildings instead of being buried below it, while on the other two managers’ farms it was due to branched water lines within buildings which, like water lines in many pig buildings, were suspended above head height under the roof, where ambient temperatures tended to increase substantially during hot days, even in buildings with insulated roofs. In the afternoon on very hot summer days, many farm managers would open a tap at the furthest point along a building’s WDS to drain warmed water and draw cooler water from underground pipes up and through the building’s system.

*Unfortunately, all our water lines are up near the roofs. We’ve done some tests in the shelters where the water doesn’t move as much. It took me 10 min after taking off a nipple to have that water cooled down enough to drink on those hot days.*

### 3.6. WDS Sanitization Practices

All managers applied rigorous cleaning/sanitization procedures to vacated pens between each batch of pigs. However, fewer than half (10/25) thoroughly cleaned/sanitized drinkers, and few cleaned/sanitized their water lines. Few managers were concerned about the impact that biofilms may have had on water quality at the drinkers and pig health. One manager who had previously worked in the poultry industry for many years remarked on this inconsistency when interviewed. Only one farm manager used a hydrogen peroxide-based disinfectant to sanitize water lines. Five managers used an organic acid product continuously to help manage biofilms, and three more managers used an acid product less frequently. Four managers flushed pipelines with plain water after each batch to remove sediments, while four managers never cleaned their water lines. Of the managers using header tanks to dose pigs, some flushed and cleaned out their header tanks after each batch; others did so less frequently or not at all.

*We’ve had some issues with slimy muck build up in the lines. Now when we empty the shed, we run the water tank down to about 500 litres of water, and we run acid through the line when the shed’s empty and clean the lines out.*

*I think about it (cleaning and sanitisation) from time to time, but that doesn’t mean action!*

## 4. Discussion

There were four main findings from this study: (1) WDSs within weaner buildings and grower/finisher buildings varied widely across farms in their configuration (looped or branched), total length (given the size of the pig accommodation area in each building), and pipe materials and diameters; (2) the WDSs within many conventional buildings and some eco-shelters were ‘over-sized’, and while this helped sustain satisfactory water flow rates from every drinker in the building 24 h per day, it would have several potentially adverse consequences; (3) in over half of the weaner buildings and one-third of the grower/finisher buildings, the number of pigs per drinker exceeded the recommended maximum, and few farms measured flow rates from drinkers quantitatively; and (4) WDS sanitization was not practiced on many farms, and few farm managers were aware of the risks to water quality and pig health posed by biofilms.

### 4.1. Looped vs. Branched WDSs

The preference for WDSs with a looped configuration in large, conventional grower/finisher buildings was understandable. While looped WDSs generally cost more to install than branched systems given that more pipe lengths are required, they offer several advantages. A looped WDS is more reliable, as water flows in two directions (rather than one, as in a branched system). Should a blockage occur at any point in the loop, all drinkers, therefore, continue to be supplied with water. With gate valves fitted along a looped WDS, sections can be isolated for repairs or maintenance without interrupting the water supply to pigs in all pens. Frictional losses are lower in a looped WDS, as part of the water flow is carried in each arm of the loop, so a smaller capacity pump is sufficient. In a looped WDS there are also fewer dead ends where water is stagnant and sediments may accumulate, as is found in municipal WDSs, in which looped systems tend to provide better residual chlorine levels [10,29,30,31]. A looped WDS may, therefore, be more effective than a branched system in transporting antimicrobials and other water-soluble additives to drinkers if build-ups of biofilms and sediments in the system are allowed to develop.

### 4.2. Over-Sized Pig Building WDSs

Participants provided data on the number of pigs and the surface area per pig in each building. The estimated length of the main pipeline of the WDS exceeded 250 m for many of the conventional buildings and some of the eco-shelters. Most of the main pipelines were 50 mm in internal diameter. Therefore, in many situations: (1) the WDS’s holding volume may be high relative to the typical ‘peaking factor’, i.e., the maximum daily usage rate divided by the average daily usage rate; (2) water must flow a considerable distance from where the pipeline enters the building to the furthest drinkers in the building; and (3) water velocities in pipe sections are likely to be very low, particularly (a) over the many hours of each day when pigs’ water usage is low to moderate; (b) in the first few weeks of occupancy by pigs, when their daily water usages are relatively low compared to the usages when they are approaching target exit bodyweight; and (c) if/when the building is not fully occupied.

We recently conducted four on-farm studies of the effects of WDS design on antimicrobial delivery to pigs that described the hydraulic performance of looped WDSs in two pig buildings with the main pipes of 50 mm internal diameter that were greater than 200 m in length [8]. The two buildings were located on a farm that participated in the survey reported here. In such buildings, very low water velocities occurred in WDS pipe sections (much lower than the minimum velocity of at least 0.5 m/sec generally specified for municipal WDSs to prevent sediment accumulation in pipes).

Farm managers viewed their large pig building WDSs as low risk, offering assurance that satisfactory water flow rates will be sustained from all drinkers 24 h per day throughout the occupancy of each batch of pigs, provided the system’s head pressure is adequate. However, an ‘over-sized’ WDS has several potential adverse consequences. First, the low velocity water flows provide more time for the water in pipes to absorb heat from the environment, leading to elevation of the temperature of the water within the system on hot days, which may lead to reduced pig growth [32,33]. Second, the low velocity water flows may facilitate the accumulation of sediment and biofilms [32]. Furthermore, when an over-sized WDS is used to administer additives to a group of pigs, its large holding volume relative to pigs’ daily water demand and very-low-water-velocity water flows through pipe sections may lead to large differences between drinkers at varying distances along the building’s WDS from the dosing pump in (1) the initial lag after commencement of dosing, before the additive first reaches the drinker, and (2) the duration over which the additive is available at the drinker. This results in between-animal variability among the group of pigs in the quantity of the additive ingested over time, and, for additives that are absorbed from the digestive tract, the systemic exposure to them [8].

### 4.3. Pigs’ Access to Water

Farm managers surveyed had placed a high priority on maintaining secure access to a source of good-quality drinking water for their stock and were acutely conscious that their pigs could not be deprived of water for more than a few hours without very serious consequences for their health and welfare. However, in buildings on most farms, water flow rates from drinkers were not measured quantitatively, and in about half of weaner buildings and a third of grower/finisher buildings the number of pigs per drinker exceeded the maxima that have been recommended [7,25,26,27,34,35,36]. When pigs are presented with water flow rates from drinkers below those recommended [4], they adapt by investing more time drinking, increasing the frequency of drinking bouts [37,38]. However, there is a limit to the ability of growing pigs to adapt to low flow rates, beyond which voluntary feed intake and daily gain are compromised. This has been shown in growers reared in hot conditions [39], although results from two studies in weaner pigs have been inconsistent [37,39].

Exceeding the recommended maxima for pigs per drinker may lead to increased competition for water between animals in each pen during periods of high water demand each day, especially in warm to hot climatic conditions. Pigs of low social rank within a small pen group (e.g., 20–40 animals) may be subjected to aggressive actions by more dominant pigs attempting to control the water supply. This may force them to alter their drinking patterns and utilize drinkers during periods of the day when they would normally be resting [7]. When pigs are reared in large pen groups (>100 animals) rather than in small pen groups, it is possible that higher maxima for pigs per drinker could be applied. Two arguments support this proposition. Firstly, like many animal species, when pigs are living in a large group they are more tolerant and less inclined to attempt to control and dominate limited resources, such as feeders and drinkers [40,41,42,43,44]. Secondly, in large group pens, active pigs have a greater free area available to them when many pigs are resting, enhancing their access to drinkers [7,45]. Our survey has indicated that a substantial proportion of Australian pig farms are rearing growing pigs in large pen groups. More research is required to determine what maxima for pigs per drinker should be applied to pigs reared in pen groups of different sizes across a range of stocking densities and environmental conditions.

Recent studies indicate that in weaners reared in small pen groups (e.g., 25 animals), providing three drinkers rather than one or two drinkers per pen results in longer, more frequent visits to drinkers, with fewer aggressive interactions [46,47]. The differing views of farm managers about the best placement and orientation of drinkers within a pen in relation to other drinkers and feeders reflected a lack of evidence-based industry guidelines on these aspects of pig water access. The use of solid partitions to create separate feeding spaces for pigs has been shown to reduce aggressive interactions between pigs [48]. However, the potential benefits of applying this concept to drinkers has not been explored. Pigs have been found to fight less frequently each day and/or with more intensity at drinkers than at feeders [6]. The use of barriers on either side of drinkers may, therefore, offer less benefit and may not significantly alter the drinking patterns of pigs.

Water wastage may be high when bite and nipple drinkers are fitted in pens and flow rates from drinkers exceed the recommended ranges [3]. Water wastage in weaner and grower/finisher buildings impacts farm water use efficiency, given that drinking water represents 80% of a pig farm’s total water use, and 75% of a farm’s water is used by growing pigs [49]. Water wastage also impacts the cost and effectiveness of in-water antimicrobial dosing and the quantities of antimicrobials that spill into the farm’s effluent system and potentially into the wider environment, increasing the risk of spread of antimicrobial-resistant genes and potential transfer to human pathogens [50].

### 4.4. WDS Sanitization Practices

The finding in our survey that a small proportion of pig farms sanitized their drinking water continuously or intermittently is a stark contrast with the findings of a recent study of Australian poultry farms, in which the majority (93%) of farms continuously sanitized their drinking water, even though they used town water, which, in most cases, is treated and sanitized prior to distribution [51]. In the poultry industry, continuous water sanitization is widely recommended as a farm biosecurity strategy, given concerns about the potential for entry of avian influenza virus and other avian pathogens through a farm’s WDS [52]. This disparity in water sanitization practices between pig farms and poultry farms is generally understandable. We found that pig farm managers had a low level of awareness of the risks that biofilms in WDSs pose to water quality and pig health. They viewed water sanitization mainly as the administration of ‘shock treatments’ between batches of pigs or, less frequently, to prevent accumulation of biofilms in WDSs [53], and had not been presented with a compelling case for continuously treating the drinking water of pigs with a disinfectant.

Pipe material is the main factor affecting the potential for formation of biofilms and also the microbial richness and diversity of biofilms [54]. In the majority of WDSs in buildings described in our survey, the main pipes were made of PVC or PE, which support less biofilm growth than pipes made of other materials, such as stainless steel, polybutylene, steel, or concrete [55,56]. Nevertheless, as part of a farm’s internal biosecurity management, water pipes and other components of the WDS of pig buildings and their in-water dosing system (including drinkers, header tanks, dosing pumps, and stock solution containers) should be cleaned and sanitized with the same vigor with which pens, feeders, and passageways are cleaned and sanitized between batches of pigs [57]. Effective elimination, or marked reduction, of potential pathogens in biofilms and free sediments depends on selection of a suitable disinfectant product, dilution of the disinfectant to the correct concentration, dispersion of the disinfectant through all pipes to all drinkers throughout the building, and provision of the specified contact time [58]. Research is required to support pig farm managers making decisions on WDS sanitization practices.

### 4.5. Use of Water-Usage Metering and Alert Systems

Many commercial systems are now available for use on pig and poultry farms that allow remote monitoring of water flow at multiple locations, report water usages in real time and generate online alarms if they deviate from a pre-determined target range, and can close valves to prevent flooding. The use of many such ‘smart’ technologies on farms offers potential benefits for animal productivity and welfare [59]. For example, automated monitoring of the drinking behavior of pigs may enable early detection of disease [60]. The farm managers we surveyed were well aware of the serious consequences of an extended period of water deprivation for pigs, especially in hot weather, and the possibility of pump failures and leaking or blocked water lines occurring outside normal daytime working hours. We therefore expected to find greater adoption of water meters and alert systems on the farms [61]. A number of factors may contribute to slow acceptance of ‘smart’ systems by a substantial proportion of animal farm managers (pigs, poultry, cattle, horses). Those suggested included skepticism about the benefits gained from using the system, a lack of trust and confidence in the system’s capabilities and performance based on previous experiences, concern about the effort required to implement and use the system, and possibly poor technology readiness of the farm [62]. Farm managers and other decision makers may also have concerns about how to collate and interpret the large quantities of data generated by one or more systems.

### 4.6. Limitations of the Study

This survey successfully integrated acquisition of quantitative and qualitative data on the design and management of WDSs in pig farm buildings. However, the survey had limitations. As explained in the Materials and Methods, our sample was not randomly selected from the population of Australian pig farm managers. We were limited in the number of parameters that we were able to investigate in the questionnaire and interviews. Our survey should be considered the first step in gaining a detailed understanding of WDSs on pig farms and the features of WDSs in weaner buildings and grower/finisher buildings that may influence the quantity and quality of water provided to growing pigs, spatially and temporally, and potentially the effectiveness of in-water dosing when practiced.

## 5. Conclusions

Over-sized and poorly designed and managed WDSs may have impacts on water provision to pigs, water quality, the effectiveness of in-water administration of antimicrobials and other additives, and, thus, on pig health and performance, while also contributing to the development of antimicrobial resistance in pig-specific bacterial pathogens.

Recommendations on water provision to growing pigs in industry guidelines currently available to pig farm managers are typically limited to (1) maxima for the number of weaners and grower/finishers per drinker; (2) heights for nipple drinkers (facing straight out from wall or angled) and bowls by pig size/bodyweight; (3) water flow rates from nipple drinkers for weaners and grower/finishers; and (4) acceptable water quality standards [26,27]. It would be valuable for industry guidelines to also provide recommendations for aspects of: WDS biofilm management/disinfection, possible modifications to existing WDSs in pig buildings to improve their hydraulic performance; monitoring of the water supply to pigs, drinkers in large pens and small pens within conventional buildings and eco-shelters, monitoring of water flow rates from nipple drinkers, water quality testing, and farm water security. (More details are available in Supplementary Materials).

## Data Availability

The data presented in this study are available on request from the corresponding author. The data are not publicly available because of the conditions of the ethics approval.

## Supplementary Materials

The following are available online at https://www.mdpi.com/article/10.3390/ani11113268/s1. S1: Survey methodology (mixed-methods approach). S2: Online questionnaire created and managed in REDCap (pdf version). S3: Semi-structured interview guide. S4: Final coding framework [NVivo] used to analyze interview transcripts. S5: Aspects of water provision to growing pigs for which recommendations should be provided in pig industry guidelines available to farm managers.

## Appendix A

**Table A1:** Demographics of the 25 pig farm managers who participated in the study.

Characteristic	Proportion of Study Participants (N)
Gender:	
Male	23/25
Female	2/25
Age:	
<25	0/25
25–34	1/25
35–44	5/25
45–54	9/25
>55	10/25
Years working in the pig industry:	
<2	0/25
2–5	1/25
6–10	3/25
>10	21/25
Years managing the current farm:	
<2	6/25
2–5	3/25
6–10	4/25
>10	12/25

**Table A2:** Characteristics of the 25 pig farms studied.

Characteristic	Proportion of Study Farms (%)
*Location [state of Australia]:*	
South Australia	9/25 (36%)
Victoria	8/25 (32%)
New South Wales	6/25 (24%)
Queensland	2/25 (8%)
Animals on farm:	
Sows, boars, and growing pigs	12/25 (48%)
Growing pigs only	13/25 (52%)
Single- or multiple-site configuration:	
Single	18/25 (72%)
Multiple	7/25 (28%)
Weaner pig buildings described in questionnaire:	
Type:	
Conventional with solid/slatted/mesh-floored pens	24/40 (60%)
Eco-shelters with straw-floor pens	16/40 (40%)
Age of buildings:	
<2 years	9/40 (22%)
3–10 years	0/40 (0%)
11–20 years	13/40 (33%)
20 years	18/40 (45%)
Grower/finisher pig buildings described in questionnaire:	
Type:	
Conventional with solid/slatted/mesh-floored pens	31/48 (65%)
Eco-shelters with straw-floor pens	17/48 (35%)
Age of buildings:	
<2 years	11/88 (12%)
3–10 years	5/88 (6%)
11–20 years	26/88 (30%)
≥20 years	46/88 (52%)
Pig flow in weaner buildings:	
All-in-all-out by room or building	38/40 (95%)
Continuous flow	2/40 (5%)
Pig flow in grower/finisher buildings:	
All-in-all-out by room or building	38/48 (79%)
Continuous flow	10/48 (21%)
