# Supplementary material for: Water Distribution Systems in Pig Farm Buildings: Critical Elements of Design and Management
Source: Animals (Basel). 2021 Nov 15;11(11):3268. doi: 10.3390/ani11113268 (PMC8614494; doi:10.3390/ani11113268)
Supplement: Supplementary file 1 [file animals-11-03268-s001.zip › S1 - Survey methodology.pdf]

Water Distribution Systems in Pig Farm Buildings: Critical Elements of Design and Management

Stephen Little, Andrew Woodward, Glenn Browning and Helen Billman-Jacobe

Survey methodology (mixed-methods approach)

The survey used a mixed-methods approach with a sequential explanatory design. Firstly, quantitative data were collected using an online questionnaire completed by each farm manager. Qualitative data were then collected using a one-on-one, semi-structured interview with each manager. Data were collected on aspects of farm WDSs as part of a larger survey that also examined in-water dosing systems and medication programmes. The questionnaire was created and managed in REDCap (Research Electronic Data Capture), a secure web-based application for building and managing online surveys (Vanderbilt University, Nashville, Tennessee, USA). Thorough pre-testing was conducted with colleagues and farm managers, and refinements were made to improve the questionnaire's clarity and ease of use before it was deployed. Participants were required to respond to all questions and asked to complete the questionnaire within two weeks of receipt using a web-link. Questionnaire responses captured in REDCap from each participant were exported into Excel, de-identified, and then analysed using the R statistical program. Data were visualised using ggplot.

Participants were interviewed by the lead author (S.L.) within four weeks of completing the online questionnaire, using an interview guide. The interview guide was also pre-tested with colleagues and farm managers. Each interview was recorded on a digital recorder (audio only) with the permission of each participant to facilitate later qualitative analysis. Interviews were transcribed verbatim from audio recordings, de-identified, entered into the qualitative data analysis software package NVivo version 12 (QSR International Pty. Ltd., Melbourne, Victoria, Australia) and openly coded and analysed using qualitative data analysis principles and thematic analysis.

Further information on the survey is provided in Supplementary Materials:
S2. Online questionnaire (REDCap)
S3: Semi-structured interview guide
S4: Final coding framework (NVivo)
