# Supplementary material for: Water Distribution Systems in Pig Farm Buildings: Critical Elements of Design and Management
Source: Animals (Basel). 2021 Nov 15;11(11):3268. doi: 10.3390/ani11113268 (PMC8614494; doi:10.3390/ani11113268)
Supplement: Supplementary file 1 [file animals-11-03268-s001.zip › S4. Final coding framework (NVIVO).pdf]

Water Distribution Systems in Pig Farm Buildings: Critical Elements of Design and Management.

Stephen Little, Andrew Woodward, Glenn Browning and Helen Billman-Jacobe

Table S4. Final coding framework (NVivo™)

Infrastructure		
	Water sources	
	On-farm water storages	
	Pipelines and fittings	
	Drinking appliances in pens	
	Strategies used to improve [changes]	
Water supply and usage by pigs		
	Day's usage in on-farm storage	
	Pigs' daily water demand	
	Peak demand periods each day	
	Water wastage by pigs	
	Water quality	
	Hygiene management	
	Problems encountered with water system and dosing equipment	
Antibiotic administration to pigs		
	Dosing equipment	
	Vet scripts and dosage calculations	
	Timing and duration of dosing events	
		Outbreaks vs. metaphylaxis
	Antibiotics and other products administered	
		In-water vs. in-feed
	Problems / preferences	
	Effectiveness of dosing events	
		Factors contributing to variability in antibiotic exposure among pigs in a group
	Strategies used to improve [changes]	
