# Supplementary material for: Water Distribution Systems in Pig Farm Buildings: Critical Elements of Design and Management
Source: Animals (Basel). 2021 Nov 15;11(11):3268. doi: 10.3390/ani11113268 (PMC8614494; doi:10.3390/ani11113268)
Supplement: Supplementary file 1 [file animals-11-03268-s001.zip › S5. Aspects of water provision for pig industry guidelines.pdf]

Water Distribution Systems in Pig Farm Buildings: Critical Elements of Design and Management
Stephen Little, Andrew Woodward, Glenn Browning and Helen Billman-Jacobe

Table S5. Aspects of water provision to growing pigs for which recommendations would be valuable in pig industry guidelines available to farm managers

Aspect of water provision:	Recommendations for:
WDS biofilm management / disinfection	• The frequencies that each building's WDS should be routinely cleaned and sanitised using a suitable disinfectant product administered through a dosing pump or header tank, as well as events that should trigger cleaning/sanitising of a WDS (e.g. contamination)<br>• The procedure and equipment for cleaning/sanitising a WDS via a dosing pump or header tank, ensuring sufficient contact time<br>• When continuous water sanitisation should be considered as a farm biosecurity strategy
Possible modifications to existing WDSs in pig buildings to improve their hydraulic performance	• Placement of non-return valves, pressure regulators, water pressure gauges, water flow meters and gate valves at appropriate points along main pipelines<br>• Removal of unnecessary fittings, constrictions and expansions along main pipelines that may be causing friction losses<br>• Replacement of pipe sections that are of excessive diameter with sections of smaller diameter<br>• Installation of additional pipe sections to a branched WDS in a large conventional building to convert it into a looped system
Monitoring of the water supply to pigs	• Installation and use of metering systems to monitor the water usage of pigs in each building and the farm's water use efficiency<br>• Installation of remote systems to alert farm staff to pump failures and leaking or burst pipes
Drinkers in large pens and small pens within conventional buildings and eco-shelters	• The maximum number of weaners and grower/finishers per drinker and optimal positioning of drinkers within large pens (>100 pigs) in relation to feeders, lying and dunging areas<br>• The maximum number of weaners and grower/finishers per drinker and optimal positioning of drinkers within small pens (20-40 pigs) in relation to feeders, lying and dunging areas<br>• Provision of a third drinker in small weaner pens, even if the number of weaner pigs per drinker is below the recommended maximum of 10, to assist newly weaned pigs to access water
Water flow rates from nipple drinkers	• The frequencies that flow rates from nipple drinkers in a building should be checked using a finger, and measured quantitatively<br>• The procedure, equipment and best time of day for quantitatively measuring water flow rates from a representative number of drinkers in a building

Aspect of water provision:	Recommendations for:
Water quality testing	• The frequency that water samples should be collected at the point of consumption by pigs (i.e. drinkers) and at its source for chemical and microbiological analysis<br>• The procedure and equipment for collecting water samples aseptically for chemical and microbiological analysis and submitting them to an accredited water testing laboratory
Farm water security	• The minimum volume of water (expressed in numbers of days of total pig usage) that should reasonably be held in on-farm water storage facilities (dams, tanks) for use if access to water from an external source is interrupted due to a power outage or mechanical failure<br>• Access to alternative water sources, should supply of the primary water source become limited at any time or its quality fall below acceptable standards for pigs
